# Prevalence and distribution of human papillomavirus genotypes in cervical intraepithelial neoplasia in China: a meta-analysis

**DOI:** 10.1007/s00404-020-05787-w

**Published:** 2020-09-10

**Authors:** Junya Zhang, Keyan Cheng, Zhilian Wang

**Affiliations:** Department of Obstetrics and Gynecology, The Second Hospital of Shanxi Medical University, 382 Wuyi Rd, Taiyuan, China

**Keywords:** Cervical intraepithelial neoplasia, Distribution, Genotype, Human papillomavirus, Meta-analysis

## Abstract

**Background and aim:**

Data on type-specific human papillomavirus (HPV) are needed to investigate HPV-based screening tests and HPV vaccines. However, Chinese relevant data are insufficient. Therefore, this meta-analysis aimed to summarize and demonstrate the prevalence and distribution of HPV genotypes in cervical intraepithelial neoplasia (CIN) and compensate for the shortage of HPV vaccines in China.

**Methods:**

The Medline, Embase, and the Cochrane Library databases, as well as references cited in the selected studies, were systematically searched for studies investigating the prevalence and distribution of HPV genotypes between January 2000 and April 2019 in China.

**Results:**

A total of 8 studies were identified, which comprised 2950 patients with CIN1 and 5393 with CIN2/3. The overall HPV infection rate was 84.37%. The HPV infection rate was significantly higher in the CIN2/3 group (87.00%) than in the CIN1 group (79.56%) (*χ*^2^ = 80.095, *P* < 0.001). The most common HPV types in CIN1 in order of decreasing prevalence were as follows: HPV52 (20.31%), HPV16 (16.81%), HPV58 (14.44%), HPV18 (6.44%), and HPV53 (5.76%). However, in the CIN2/3 group, HPV16 (45.69%) was the predominant type, followed by HPV58 (15.50%), HPV52 (11.74%), HPV33 (9.35%), and HPV31 (4.34%).

**Conclusions:**

This study suggested that HPV16, HPV52, and HPV58 were the top three types of CIN in China. The findings might provide a reference for future HPV-based cervical cancer screening tests, treatment of HPV infection, and application of HPV vaccines in China.

## Introduction

Of the 570,000 new patients of cervical cancer reported worldwide in 2018, more than 310,000 patients died [1]. The International Agency for Research on Cancer combined 22 countries to implement a large-scale epidemiological study in 1999. Human papillomavirus (HPV) was identified in 99.7% of patients with cervical cancer [2]. A significant discovery by Hausen indicated that persistent infection of high-risk human papillomavirus (HR-HPV) would result in cervical lesions [3]. Cervical intraepithelial neoplasia (CIN) is a preinvasive form of cervical cancer. Persistent HPV infection may significantly increase the risk of CIN progression to cervical cancer [4]. Thus, cervical cancer has become the only gynecological malignant tumor with clear etiology in the world.

The genome of HPV comprises a double-strand circular DNA of 8000 base pairs. More than 100 types of HPVs have been identified so far. They are divided into two categories: HR-HPV and low-risk HPV (LR-HPV). Furthermore, 80% of women are infected with HPV in their lifetime; however, around 90% of infections disappear [5]. It is generally accepted that HPV16 is the major high-risk genotype in the world, while HPV52 and HPV58 are common in Asia [6]. The prevalence of HPV genotype is associated with distinct races, regions, and customs [7, 8].

At present, the use of HPV vaccines is one of the most important ways to refrain from HPV infection [9]. However, the existing HPV vaccines have been prepared based on the epidemiologic characterization of HPV infection in Western countries. Hence, the development and application of HPV vaccines in China face extraordinary challenges. Therefore, this meta-analysis was performed to summarize and demonstrate the prevalence and distribution of HPV genotypes in CIN and compensate for the shortage of HPV vaccines in China.

## Materials and methods

### Study selection

The Medline, Embase, and the Cochrane Library databases, as well as references cited in the selected studies, were systematically searched for studies investigating the prevalence and distribution of HPV genotypes between January 2000 and April 2019 in China. The key search items included “cervical intraepithelial neoplasia,” “human papillomavirus,” “polymerase chain reaction,” “epidemiology,” “genotype,” “China,” “female,” and so forth. The meta-analysis was restricted to studies published between January 2000 and April 2019. The analysis had no language restrictions.

### Inclusion and exclusion criteria

The inclusion criteria were as follows: (1) research related to opportunistic screening in hospitals, (2) CIN1, CIN2, and CIN3 diagnosed by cervical histopathology, (3) each targeted study contained a minimum of 20 CIN cases, (4) HPV-DNA detected by polymerase chain reaction (PCR), and (5) at least 10 identified HPV types. The exclusion criteria were as follows: (1) no pathological result found, (2) the grouping included cervical cancer, and (3) the requisite information could not be accessed.

### Data abstraction

The data from all the studies were collected and checked simultaneously by the researchers. The main extracted information included the first author name, reference, research location, HPV-DNA source, PCR primers, sample capacity, and HPV prevalence (see Table 4 in “Appendix”).

### Statistical analysis

The rate and the confidence interval (CI) of more than 95% were used for the correlation analysis. The statistical analysis used the CI and heterogeneity test combined with the rate of logit transformation. Review Manager 5.3 was used for the meta-analysis of the correlation between HPV infection and cervical lesions. When *P* was > 0.05 and *I*^2^ was < 50%, the fixed-effects model was used. When *P* was < 0.05 and *I*^2^ was > 50%, statistical heterogeneity was observed. The source of heterogeneity was analyzed and eliminated. If heterogeneity still existed, the meta-analysis was carried out using the random-effects model. The potential source of bias was assessed by drawing funnel plots.

## Results

**Fig. 1:**
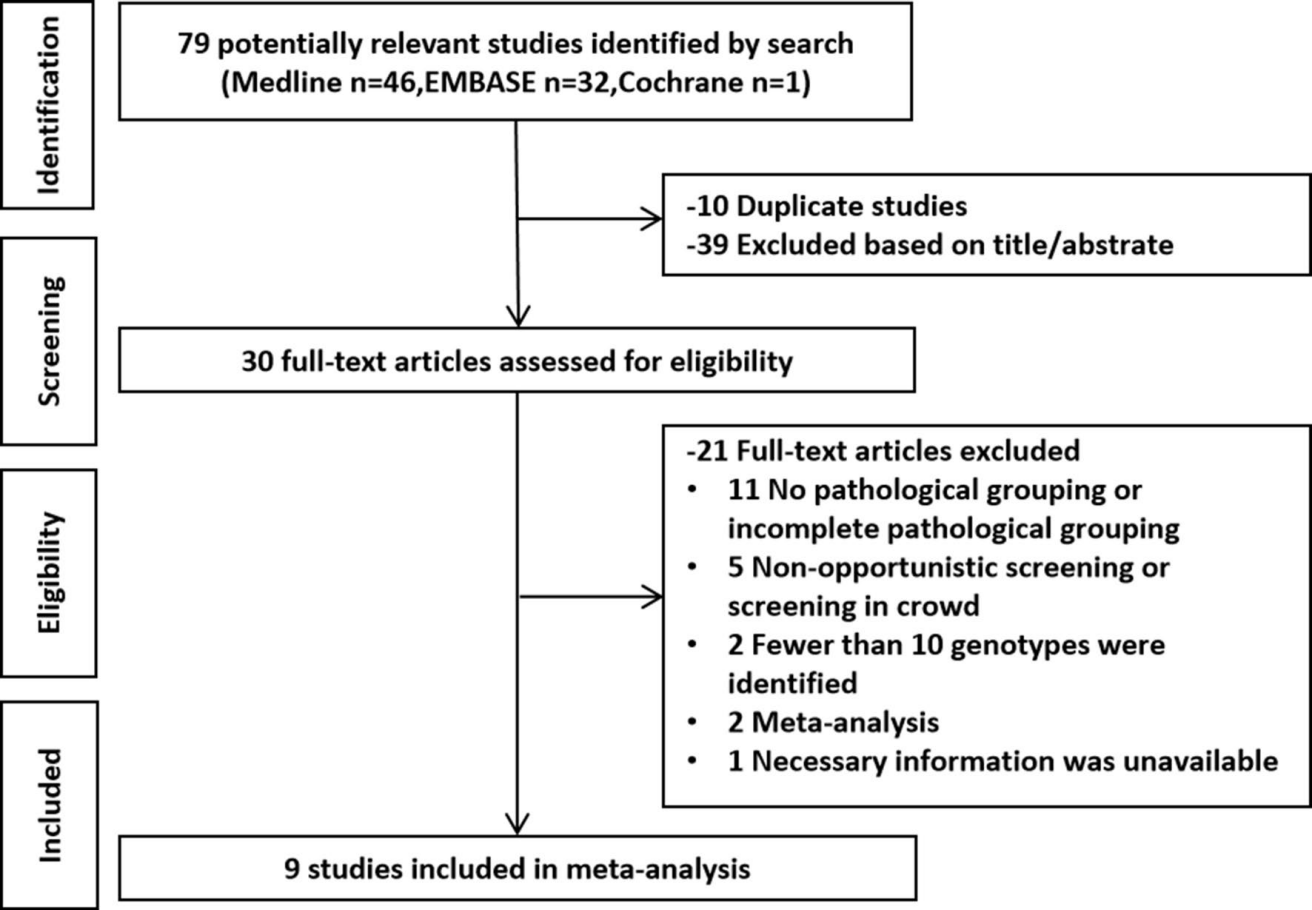
Flow diagram for the identification of studies for meta-analysis

A total of 79 potentially related studies were identified by screening Medline, Embase, and the Cochrane Library. Of these, 10 studies presented duplicate reports and 40 had nothing to do with the epidemiological characteristics of HPV. Ultimately, nine studies were included in the meta-analysis after full-text screening [10–18]. The literature screening process is shown in Fig. 1.

A meta-analysis of total HPV infection rates was carried out in the CIN2/3 and CIN1 groups. The random-effects model was used because of significant heterogeneity (*I*^2^ = 97%, *P* < 0.00001). Unfortunately, an obvious bias was found. Subsequently, the infection rate of the most representative HPV16 was explored in the meta-analysis of CIN2/3 and CIN1 groups. As a result, a moderate heterogeneity (*I*^2^ = 51%, *P* = 0.04) was noted, and the bias decreased significantly.

**Fig. 2:**
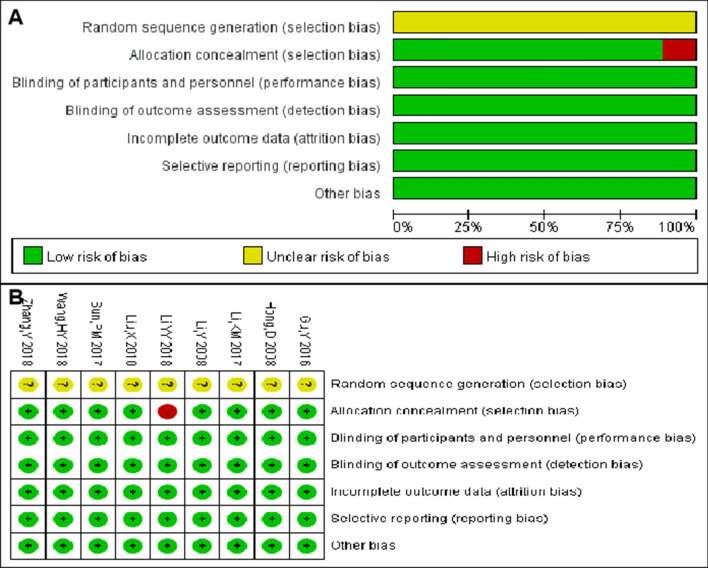
**a** Risk of bias for the included studies, and **b** risk-of-bias summary for the included studies

**Fig. 3:**
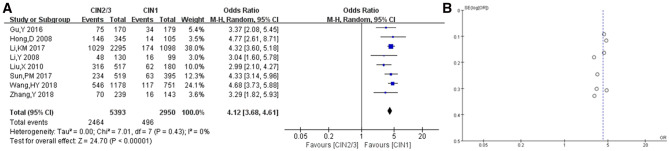
**a** Forest plot depiction of HPV-16 infection rates and odds ratios compared between the CIN2/3 and CIN1 groups (eight studies), and **b** funnel plot for the assessment of publication bias regarding the relationship between HPV and cervical lesions (eight studies)

Subsequently, the risk of bias was assessed one by one, and the risk of bias and the bias summary among the included studies are shown in Fig. 2. The selected studies were cohort or case–control studies. None of them referred to the principle of randomness. A high risk of bias was observed only in the study by Li in 2018 [14], which was, therefore, examined again in detail. The study showed that the samples in Yunnan province were divided into Han ethnic and non-Han ethnic groups. Also, a small difference was found in the prevalence of HPV between the two groups. However, in other studies, the participants did not belong to the areas, where ethnic minorities gathered; most of them were of Han nationality. Consequently, the study by Li was excluded. The HPV16 infection rate was compared between the CIN2/3 and CIN1 groups in the finally selected eight studies. No heterogeneity (*I*^2^ = 0%, *P* = 0.43) or apparent bias was found (Fig. 3).

**Fig. 4:**
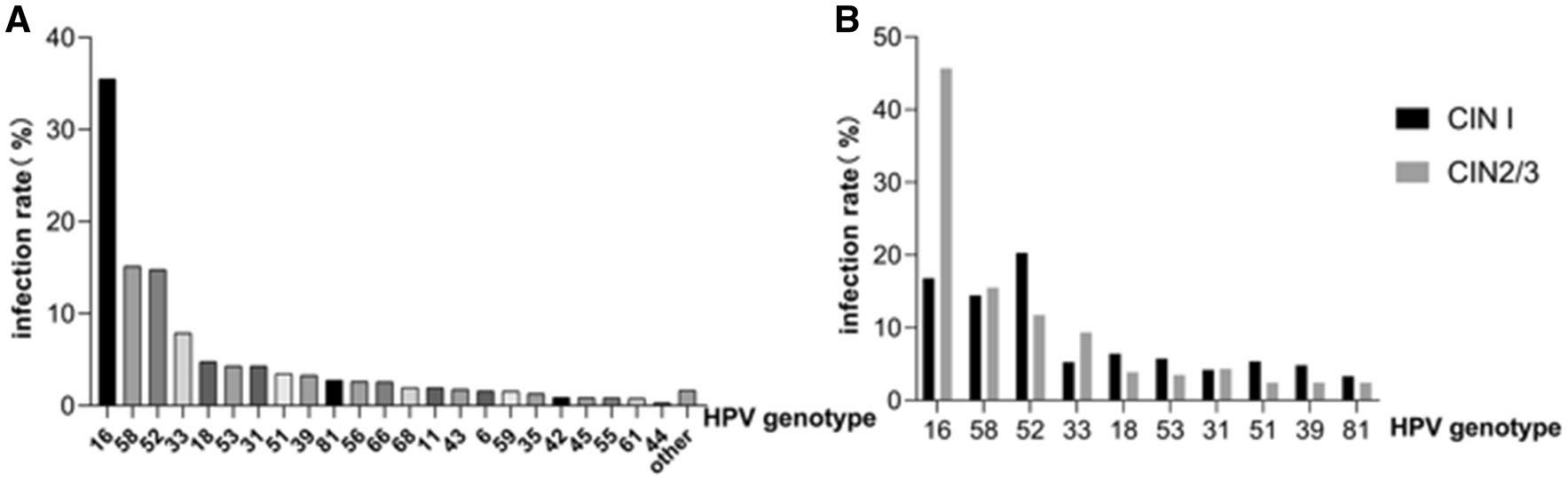
**a** Distribution of HPV genotypes in 8343 samples, and **b** distribution of HPV genotypes in different pathological groups

The selected studies summarized the distribution of HPV genotypes in 8343 patients with CIN. The overall HPV infection rate was 84.37%. The prevalence of HPV genotypes is shown in Table 1: HPV16 (34.56–36.61%), HPV58 (14.36–15.90%), HPV52 (14.01–15.53%), HPV33 (7.32–8.48%), HPV18 (4.32–5.24%), HPV53 (3.87–4.74%), HPV31 (3.86–4.73%), HPV51 (3.08–3.87%), HPV39 (2.90–3.67%), and HPV81 (2.39–3.10%) (Fig. 4a).

**Table 1:** Prevalence of HPV genotypes in cervical lesion. (*n* = 8343)

HPV genotype	*N*	Infection rate (%)	95% CI
Overall	7039	84.37	83.59–85.15
HR-HPV			
16	2960	35.48	34.56–36.61
52	1232	14.77	14.01–15.53
58	1262	15.13	14.36–15.90
18	399	4.78	4.32–5.24
53	359	4.30	3.87–4.74
68	164	1.97	1.67–2.26
33	659	7.90	7.32–8.48
39	274	3.28	2.90–3.67
51	290	3.48	3.08–3.87
56	219	2.62	2.28–2.97
45	73	0.87	0.68–1.07
81	229	2.74	2.39–3.10
35	109	1.31	1.06–1.55
66	214	2.57	2.23–2.90
LR-HPV			
6	132	1.58	1.31–1.85
31	358	4.29	3.86–4.73
43	148	1.77	1.49–2.06
44	24	0.29	0.17–0.40
59	131	1.57	1.30–1.84
11	164	1.97	1.67–2.26
42	74	0.89	0.69–1.09
55	69	0.83	0.63–1.02
61	64	0.77	0.58–0.95
Other	141	1.69	1.41–1.97

*HR-HPV* high-risk human papillomavirus, *LR-HPV* low risk human papillomavirus

The samples included 2950 patients with CIN1 and 5393 with CIN2/3. The HPV infection rate was significantly higher in the CIN2/3 group (87.00%) than in the CIN1 group (79.56%) (*χ*^2^ = 80.095 and *P* < 0.001). In the CIN1 group, HPV52 (20.31%), HPV16 (16.81%), HPV58 (14.44%), HPV18 (6.44%), and HPV53 (5.76%) were the most common types. Yet, in the CIN2/3 group, HPV16 (45.69%), HPV58 (15.50%), HPV52 (11.74%), HPV33 (9.35%), and HPV31 (4.34%) were the most common types. The infection rate of each type was dramatically different between the two groups (Table 2; Fig. 4b).

**Table 2:** HPV genotypes distribution in different degree of cervical lesion (*n* = 8343)

HPV genotype	CIN1 (*n* = 2950) *N* (%)	CIN2/3 (*n* = 5393) *N* (%)	*χ*^2^	*P*
合计	2347 (79.56)	4692 (87.00)	80.095	< 0.001
HR-HPV				
16	496 (16.81)	2464 (45.69)	694.563	< 0.001
52	599 (20.31)	633 (11.74)	111.213	< 0.001
58	426 (14.44)	836 (15.50)	1.672	0.196
18	190 (6.44)	209 (3.88)	27.557	< 0.001
53	170 (5.76)	189 (3.51)	23.614	< 0.001
68	86 (2.92)	78 (1.45)	21.352	< 0.001
33	155 (5.25)	504 (9.35)	43.874	< 0.001
39	143 (4.85)	131 (2.43)	35.112	< 0.001
51	158 (5.36)	132 (2.45)	48.073	< 0.001
56	127 (4.31)	92 (1.71)	50.400	< 0.001
45	24 (0.81)	49 (0.91)	0.199	0.656
81	98 (3.32)	131 (2.43)	5.696	0.017
35	50 (1.69)	59 (1.09)	5.340	0.021
66	115 (3.90)	99 (1.84)	32.460	< 0.001
LR-HPV				
6	62 (2.10)	70 (1.30)	7.911	0.005
31	124 (4.20)	234 (4.34)	0.085	0.770
43	84 (2.85)	64 (1.19)	30.183	< 0.001
44	11 (0.37)	13 (0.24)	1.155	0.282
59	69 (2.34)	62 (1.15)	17.453	< 0.001
11	78 (2.64)	86 (1.59)	10.897	0.001
42	38 (1.29)	36 (0.67)	8.274	0.004
55	45 (1.53)	24 (0.45)	27.138	< 0.001
61	28 (0.95)	36 (0.67)	1.987	0.159
Other	40 (1.36)	101 (1.87)	3.066	0.080

*HR-HPV* high-risk human papillomavirus, *LR-HPV* low risk human papillomavirus, *CIN* cervical intraepithelial neoplasia

Five out of eight studies reported HPV infection rates of single and multiple types. Four studies were further grouped pathologically. The single HPV infection rate was higher in the CIN2/3 group than in the CIN1 group (*χ*^2^ = 26.444, *P* < 0.001). However, no obvious difference was found in the multiple HPV infection rate between the two groups (Table 3).

**Table 3:** Comparison between single and multiple HPV infections in cervical lesions of different degrees, *n* (%)

	Total^a^	CIN1^b^	CIN2/3^b^	*χ*^2^	*P*
*N*	5368	742	1233		
HPV (%)	4137 (77.07)	454 (61.19)	881 (71.45)	22.287	< 0.001
Single (%)	3051 (56.84)	296 (39.89)	643 (52.15)	27.905	< 0.001
Multiple (%)	1086 (20.23)	158 (21.29)	238 (19.30)	1.146	0.284

^a^Indicates an additional study besides b

## Discussion

This study systematically reviewed eight published studies on the distribution of HPV genotype in China, controlled the heterogeneity of these studies, and improved the accuracy of HPV genotype description for Chinese women with CIN. According to the study by Li, the HR-HPV infection rate in the CIN1 group was 59.5% (95% CI 52.7–66.4) [7], which was lower than the values obtained in the present study (67.72%). Taking into account More than 20 high-risk and low-risk HPV genotypes, the total HPV infection rate was found to be higher in the present study compared with other studies. In 2018, a Chinese study reported that the HPV infection rate in the CIN2/3 group was 79.9–87.6% [19], consistent with the result of the present meta-analysis.

HPV16 has the highest incidence of cervical cancer, while the distribution of other types varies regionally in different countries [20]. Some meta-analyses demonstrated the association of HPV16 and HPV18 with invasive cervical cancer globally, including Asia [21, 22]. However, in China, the most common HPV types in order of decreasing prevalence were as follows: HPV16, HPV58, HPV52, HPV18, and HPV33 [7]. The order in the present meta-analysis was as follows: HPV16, HPV58, HPV52, HPV33, and HPV18. A European study found that the three top HPV genotypes in the CIN2/3 group were HPV16 (48.1%), HPV31 (13.9%), and HPV33 (9.9%) [23]. In the present meta-analysis, HPV16 was still the most common type among women with CIN2/3, followed by HPV58 and HPV52. Hence, the distribution of HPV genotypes varied in different regions and cervical lesions, indicating that the application of HPV vaccines should adapt to local conditions. A large-scale epidemiological survey of HPV in 51,345 women in China showed that HPV52, HPV16, and HPV18 were the 3 most common types [24]. In the present meta-analysis, the top five genotypes with the highest infection rate in the CIN1 group were HPV52, HPV16, HPV58, HPV18, and HPV53. The top two HPV types were identical to those in the aforementioned survey. Previous studies on HPV genotypes of 1387 women with CIN2/3 in Shanxi province showed that the most prevalent types were HPV16 (59.3%), HPV58 (14.4%), and HPV33 (10.0%) [25]. In the present meta-analysis, HPV16, HPV58, HPV52, HPV33, and HPV31 were the most common types in the CIN2/3 group. The results were consistent with previous findings.

Therefore, it was concluded that the main types of HPV infection in the CIN1 and CIN2/3 groups were different. Furthermore, the infection rate of each genotype was markedly diverse for cervical lesions of different degrees. It was confirmed again that the most common HPV genotypes among Chinese women were different from those in non-Asian countries. According to the “2012 Updated Consensus Guidelines for the Management of Abnormal Cervical Cancer Screening Tests and Cancer Precursors,” HPV16- or HPV18-infected women were supposed to first undergo colposcopy despite negative results on cytologic evaluation [26]. However, in the present study, HPV52 and HPV58 were far more common than HPV18, especially in women with CIN2/3, indicating that colposcopy was needed for women with normal results on cytologic evaluation but with HPV52 or HPV58 infection. Hence, it is important for China to improve the strategy of cervical cancer screening and treatment.

The safety and efficacy of HPV vaccines have been confirmed [27]. At present, three kinds of HPV vaccines are available: Cervarix, Gardasil, and Gardasil 9. The Cervarix vaccine prevents mainly the infection caused by HPV16 and HPV18. The Gardasil vaccine is used primarily for resisting HPV6, HPV11, HPV16, and HPV18. The Gardasil 9 vaccine affects HPV6, HPV11, HPV16, HPV18, HPV31, HPV33, HPV45, HPV52, and HPV58. The distribution of HPV types in China includes HPV16, HPV58, HPV52, HPV33, HPV18, HPV53, HPV31, HPV51, HPV39, and HPV81 (in order of decreasing prevalence). Cervarix and Gardasil have limited functions, with no effects on HPV52, HPV58, and HPV33. Gardasil 9 offers sufficient protection against HPV infection. The HPV53 infection rate is 3.87–4.74%, ranking sixth for CIN in China. The CIN1 infection rate ranks fifth. However, universal HPV vaccines are useless against HPV53. Therefore, the development of new vaccines to prevent HPV infection based on the prevalence and distribution characteristics of HPV genotypes in China is of great significance.

The types of HPV infection are diverse depending on the difference in the susceptibility of different individuals. Several studies showed that a single HPV infection was dominant, while multiple HPV infections were prevalent in large populations [28, 29]. Researchers have different opinions on the relationship between multiple HPV infections and cervical lesions. A study showed that multiple HPV infections were a common phenomenon and did not increase the risk of progression of CIN. In addition, they might cause more effective immune responses due to competition, reducing the incidence of high-grade CIN [30]. Other studies suggested that multiple HPV infections had a synergistic effect on the development of cervical cancer [31]. Compared with single HPV infection, patients with CIN1 having multiple HPV infections had a higher risk of developing into CIN2 + [32, 33]. Zhao argued that the HPV infection rate increased with the increase in the CIN grade (*χ*^2^ = 62.875, 22.113, *P* < 0.001) [34]. In the present study, the single HPV infection rate was remarkably higher in the CIN2/3 group than in the CIN1 group, while the multiple HPV infection rate was slightly higher in the CIN2/3 group than in the CIN1 group. Another study showed that a persistent HPV infection, mainly caused by polytypic HPV and HR-HPV [35], was an important factor in the development of CIN [36]. Therefore, exploring multiple HPV infections offers a reference for the development of effective HPV vaccines.

The present study had several limitations. First, HPV results were influenced by the DNA source and detection techniques. Second, despite the detailed description of type-specific HPV, the analysis of multiple HPV infections was simple. The influence of co-infection with different genotypes on CIN was of great significance. Third, it was meaningful to group cases by region or ethnicity. Last but not least, the study was limited to the investigation of CIN and lacked data on healthy people and patients with cervical cancer. Therefore, more detailed investigations are needed in the future.

In conclusion, the distribution of papillomavirus genotypes in China basically resembles that in other parts of the world. HPV16, HPV52, and HPV58 are the most common types of CIN in China. At present, the universal HPV Gardasil 9 vaccine is effective against most of the HPV types worldwide. However, the existing HPV vaccines do not play any role against HPV53, which has a high prevalence rate. This study provided a reference for future HPV-based cervical cancer screening tests, treatment of HPV infection, and application of HPV vaccines in China.

## Appendix

See Table 4.

**Table 4:** Study methods and type-specific prevalence of human papillomavirus by study are summarized

First author	Reference	Province	HPV DNA source	PCR primers used to identify all HPV + ve	No. cases	CIN1/CIN2/3	HPV prevalence (% of all cases tested)																										
							Any	16	18	52	58	53	68	33	39	51	56	45	81	35	66	6	31	43	44	59	11	42	55	61	Oth-er	Single	Multiple
Gu, Y	[10] Oncotarget (2016)	Shanghai	Exfol. cells		349	179/170	72.5	43.8	9.2	28.5	30.5	8.8	7.2		12.9	9.6	2.4	3.2	1.6	3.2	5.2		10.8			2.4							
Hong, D	[11] Int J Gynecol Cancer (2008)	Zhejiang	Exfol. cells	MY09/11	450	105/345	84.9	35.6	5.1	3.1	18.0	15.6	0.8	10.0	1.6	0.6	0.6	1.8	0.1		0.1	0.1	4.2			0.6	2.2			1.6	3.1	75.3	9.6
Li, KM	[12] Medline (2017)	Sichuan	Exfol. cells		3393	1098/2295	82.6	35.5	4.4	15.0	12.0	4.3	0.3	6.2	3.7	4.2	3.4	0.7	2	13.2	2.6	1.3	2.1	2.2	0.5	2.0	1.4	0.8	2	1.7	2.7	62.2	20.3
Li, Y	[13] Cancer Genetics and Cytogenetics (2008)	Beijing	Exfol. cells	MY09/11	229	99/130	84.3	27.9	7.9	13.5	14.4	6.1	1.3	6.6	2.2	3.9	2.2	0.9		1.3	3.5	5.2	6.1		0.9	5.2	3.5	0.4			2.6	56.3	27.9
Li, YY	[14] Journal of Infection and Public Health (2018)	Yunnan	Fixed biopsies	MY09/11,GP5/6	443	205/238	75.6	19.2	5.2	20.1	12.9	5.4	3.8	5.6	4.3	3.6	1.8	1.4	2.9	1.1	2.0	1.1	1.1	0.9	0.5	1.1	0.9	0.2	0.2	0.2	4.5	55.3	20.3
Liu, X	[15] Int J Gynecol Cancer (2010)	Liaoning	Exfol. cells	PGMY09/PGMY11	697	180/517	84.6	54.2	2.2	7.2	14.1	3.4	2.3	12.1	1.6	1.0	0.1	1.3	3	0.7	1.9	2.9	7.9	0.1	0.6	0.9	2.7	0.1					
Sun, PM	[16] J Gynecol Oncol (2017)	Fujian	Exfol. cells	PGMY09/PGMY11	914	395/519	86.7	32.5	7.2	18.6	16.3	4.2	3.6	8.4	2.6	4.7	4.4	1.8	1.3	1.6	3.3	1.9	4.7	6.8		3.4	2.5	3.3			2.5	26.4	27.5
Wang, HY	[17] BMC Cancer (2018)	Zhejiang	Exfol. cells		1929	751/1178	91.2	34.4	4.8	18.2	20.2	5.2	3.9	10.9	2.9	2.5	2.1		5.3	1.2	2.5	1.1	6.3				1.5	0.7					
Zhang, Y	[18] Int J Clin Exp Med (2018)	Sichuan	Exfol. cells		382	143/239	70.2	22.5	2.8	8.9	9.4	1.8	1	4.7	3.7	3.1	1.6	1.6	4.2	2.6	2.6	3.1	1.3	2.6	0.0	1	7.1	0.3			2.1	65.4	9.9

## References

[1] Bray F, Ferlay J, Soerjomataram I, Siegel RL, Torre LA, Jemal A (2018). Global cancer statistics 2018: GLOBOCAN estimates of incidence and mortality worldwide for 36 cancers in 185 countries. CA Cancer J Clin.

[2] Walboomers JM, Jacobs MV, Manos MM, Bosch FX, Kummer JA, Shah KV (1999). Human papillomavirus is a necessary cause of invasive cervical cancer worldwide. J Pathol.

[3] zur Hausen H (2009). 1372 Cervical cancer: From basic studies to papillomavirus vaccination. Int J Gynecol Obstet.

[4] Li H, Wang X, Geng J, Zhao X (2015). Clinical study of styping detection of human papillomavirus (HPV) infection with microarray from paraffinembedded specimens of cervical cancer and precursor lesions. J Nanosci Nanotechnol.

[5] Gong P, Wang Z, Geng J, Tan X (2017). Comparative study on detection and typing of human papillomavirus (HPV) infection with microarray using paraffin-embedded specimens from squamous cell carcinoma and cervical precursor lesions. J Nanosci Nanotechnol.

[6] Bosch FX, Burchell AN, Schiffman M, Giuliano AR, de Sanjose S, Bruni L (2008). Epidemiology and natural history of human papillomavirus infections and type-specific implications in cervical neoplasia. Vaccine.

[7] Li K, Li Q, Song L, Wang D, Yin R (2019). The distribution and prevalence of human papillomavirus in women in mainland China. Cancer.

[8] Guan P, Howell-Jones R, Li N, Bruni L, de Sanjosé S, Franceschi S (2012). Human papillomavirus types in 115,789 HPV-positive women: a meta-analysis from cervical infection to cancer. Int J Cancer.

[9] Giuliano AR, Kreimer AR, de Sanjose S (2015). The beginning of the end: vaccine prevention of HPV-driven cancers. J Natl Cancer Inst.

[10] Gu Y, Ma C, Zou J, Zhu Y, Yang R, Xu Y (2016). Prevalence characteristics of high-risk human papillomaviruses in women living in Shanghai with cervical precancerous lesions and cancer. Oncotarget.

[11] Hong D, Ye F, Chen H, Lü W, Cheng Q, Hu Y (2008). Distribution of human papillomavirus genotypes in the patients with cervical carcinoma and its precursors in Zhejiang Province, China. Int J Gynecol Cancer.

[12] Li K, Yin R, Li Q, Wang D (2017). Analysis of HPV distribution in patients with cervical precancerous lesions in Western China. Medicine (Baltimore).

[13] Li Y, Wang Y, Jia C, Ma Y, Lan Y, Wang S (2008). Detection of human papillomavirus genotypes with liquid bead microarray in cervical lesions of northern Chinese patients. Cancer Genet Cytogenet.

[14] Yuanyue L, Baloch Z, Yasmeen N, Tao Y, Xiaomei W, Xueshan X (2018). The distribution of human papillomavirus genotypes in cervical cancer and intraepithelial neoplasia lesions among Chinese women in Yunnan Province. J Infect Public Health.

[15] Liu X, Zhang S, Ruan Q, Ji Y, Ma L, Zhang Y (2010). Prevalence and type distribution of human papillomavirus in women with cervical lesions in Liaoning Province, China. Int J Gynecol Cancer.

[16] Sun P, Song Y, Ruan G, Mao X, Kang Y, Dong B (2017). Clinical validation of the PCR-reverse dot blot human papillomavirus genotyping test in cervical lesions from Chinese women in the Fujian province: a hospital-based population study. J Gynecol Oncol.

[17] Wang H, Cheng X, Ye J, Xu X, Hong Y, Sui L (2018). Distribution of human papilloma virus genotype prevalence in invasive cervical carcinomas and precancerous lesions in the Yangtze River Delta area, China. BMC Cancer.

[18] Zhang Y, Luo X, Ruan SB, Xiao XL, Tang XQ, Yang ZH (2018). Human papillomavirus (HPV) genotype distribution in women with cervical lesions in Southern Sichuan of China. Int J Clin Exp Med.

[19] Xu H, Wang K, Feng X, Dong S, Lin A, Zheng L (2018). Prevalence of human papillomavirus genotypes and relative risk of cervical cancer in China: a systematic review and metaanalysis. Oncotarget.

[20] Li N, Franceschi S, Howell-Jones R, Snijders PJ, Clifford GM (2011). Human papillomavirus type distribution in 30,848 invasive cervical cancers worldwide: variation by geographical region, histological type and year of publication. Int J Cancer.

[21] Clifford GM, Smith JS, Plummer M, Muñoz N, Franceschi S (2003). Human papillomavirus types in invasive cervical cancer worldwide: a meta-analysis. Br J Cancer.

[22] Smith JS, Lindsay L, Hoots B, Keys J, Franceschi S, Winer R, Clifford GM (2007). Human papillomavirus type distribution in invasive cervical cancer and high-grade cervical lesions: a meta-analysis update. Int J Cancer.

[23] Tjalma WA, Fiander A, Reich O, Powell N, Nowakowski AM, Kirschner B (2013). Differences in human papillomavirus type distribution in high-grade cervical intraepithelial neoplasia and invasive cervical cancer in Europe. Int J Cancer.

[24] Zeng Z, Yang H, Li Z, He X, Griffith CC, Chen X (2016). Prevalence and genotype distribution of HPV infection in china: analysis of 51,345 HPV genotyping results from China’s largest CAP certified laboratory. J Cancer.

[25] Wang Z, Li Z, Li J, Wang C, Wang W, Hao M (2018). Prevalence and distribution of HPV genotypes in 1387 women with cervical intraepithelial neoplasia 2/3 in Shanxi province, China. J Cancer.

[26] Massad LS, Einstein MH, Huh WK, Katki HA, Kinney WK, Schiffman M (2013). 2012 updated consensus guidelines for the management of abnormal cervical cancer screening tests and cancer precursors. Obstet Gynecol.

[27] Dilley S, Miller K, Huh W (2018). HPV vaccination. Gynecol Oncol.

[28] Hajia M, Sohrabi A (2018). Possible synergistic interactions among multiple HPV genotypes in women suffering from genital neoplasia. Asian Pac J Cancer Prev.

[29] Sohrabi A, Hajia M, Jamali F, Kharazi F (2017). Is incidence of multiple HPV genotypes rising in genital infections?. J Infect Public Health.

[30] Salazar KL, Zhou HS, Xu J, Peterson LE, Schwartz MR, Mody DR (2015). Multiple human papilloma virus infections and their impact on the development of high-risk cervical lesions. Acta Cytol.

[31] Trottier H, Mahmud S, Costa MC, Sobrinho JP, Duarte-Franco E, Rohan TE (2006). Human papillomavirus infections with multiple types and risk of cervical neoplasia. Obstet Gynecol Surv.

[32] Xu HH, Lin A, Chen YH, Dong SS, Shi WW, Yu JZ (2017). Prevalence characteristics of cervical human papillomavirus (HPV) genotypes in the Taizhou area, China: a cross-sectional study of 37 967 women from the general population. BMJ Open.

[33] Chaturvedi AK, Katki HA, Hildesheim A, Rodríguez AC, Quint W, Schiffman M (2011). Human papillomavirus infection with multiple types: pattern of coinfection and risk of cervical disease. J Infect Dis.

[34] Zhao Y, Zhao F, Hu S, Chen W, Chen F, Cui J (2015). Multi-center cross-sectional study on type-specific human papillomavirus infection among Chinese women. Zhonghua Liu Xing Bing Xue Za Zhi.

[35] Xiao SS, Fan JL, He SL, Li YR, Wang LY, Yu KN (2016). Analysis of human papillomavirus infection in 16,320 patients from a gynecology clinic in Central South China. J Low Genit Tract Dis.

[36] Kang TS, Wang W, Zhong HJ, Liang JX, Ko CN, Lu JJ (2017). A rhodium(III)-based inhibitor of autotaxin with antiproliferative activity. Biochim Biophys Acta Gen Subj.

